# The Bacterial Metabolite Lndole-3-Propionic Acid Alleviates Acetaminophen-Induced Acute Liver Injury Partly through the Aryl Hydrocarbon Receptor Pathway

**DOI:** 10.4014/jmb.2604.04031

**Published:** 2026-07-30

**Authors:** Bo Zhou, Xiushang Ye, Yuan Tian, Xintong Wang, Xiaozhang Xu, Yi Ren

**Affiliations:** 1 Department of General Surgery, Ningbo Medical Center Li HuiLi Hospital (The Affiliated Lihuili Hospital of Ningbo University), 315040, Ningbo, Zhejiang, P. R. China; 2 Department of Breast and Thyroid Surgery, The Affiliated Huaian No.1 People's Hospital of Nanjing Medical University, Northern Jiangsu Institute of Clinical Medicine, Nanjing Medical University, 223300, Huaian, Jiangsu, P. R. China; 3 Department of General Surgery, Ninghai Chengguan Hospital (Ninghai Branch of Li Huili Hospital), 315000, Ningbo, Zhejiang, P. R. China; 4 Department of Breast and Thyroid Surgery, The Huaian Clinical College of Xuzhou Medical University, Huai'an, 223300, Huaian, Jiangsu, P. R. China

**Keywords:** Acetaminophen, Acute liver injury, Indole-3-propionic Acid, Aryl hydrocarbon receptors

## Abstract

Acetaminophen (APAP)-induced liver injury is a major cause of acute liver injury (ALI). Indole-3-propionic acid (IPA), a gut microbiota–derived tryptophan metabolite with potent anti-inflammatory and antioxidant properties, has not been fully explored for its therapeutic potential in APAP-induced ALI. APAP-induced liver injury is often accompanied by dysregulation of both the gut microbiota and the intrahepatic immune system. In our study, IPA levels were markedly reduced in the ALI model, reflecting impaired gut tryptophan metabolism. *In vitro* and *in vivo* experiments demonstrated that IPA mitigates cytotoxicity, reduces hepatocyte apoptosis, and alleviates liver inflammation, thereby conferring significant hepatoprotective effects. The aryl hydrocarbon receptor (AhR), a key mediator of IPA’s effects, was substantially downregulated in APAP-induced ALI and negatively correlated with serum AST and ALT levels, suggesting that AhR may serve as a potential early biomarker for APAP-induced hepatotoxicity. Notably, IPA treatment restored hepatic and serum AhR expression and attenuated liver injury. Collectively, these findings underscore the critical role of IPA in modulating AhR signaling and highlight its therapeutic potential in APAP-induced ALI.

## Introduction

APAP is a commonly used analgesic and antipyretic that is safe at therapeutic concentrations, but can cause severe acute liver damage when taken in excess, which is also a common public health concern [1]. APAP is rapidly absorbed by the intestine and catalyzed by liver cytochrome P450 2E1 (CYP2E1) to N-acetyl-p-benzoquinone imine (NAPQI), which is eliminated by binding to glutathione (GSH); however, excessive NAPQI can lead to oxidative stress and necrosis of hepatocytes [2]. Although numerous studies over the past 40 years have shown the beneficial effects of many compounds, the U.S. Food and Drug Administration recommends N-acetylcysteine as the only treatment option for patients with APAP overdose. However, this drug has several limitations, including adverse reactions and a narrow therapeutic window [3]. Therefore, it is necessary to identify more effective treatments for APAP-induced liver injury.

The gut microbiota and their metabolites have been shown to regulate many extraintestinal organ diseases, including liver injury [4]. The gut-liver axis serves as a bridge for the interaction between the gut microbiota and the liver. Microbially derived granisetron has been reported to effectively reduce liver damage caused by sepsis [5]. Changes in the gut microbiota affect APAP-induced liver injury through various pathways, such as the transfer of gut bacteria to the liver and promotion of liver inflammation during CCl4-induced fibrosis[4]. In addition, saturated fatty acids produced by the gut microbiota protect against alcohol-induced liver damage [6]. Indole-3-propionic acid (IPA) is a product of tryptophan metabolism by the gut microbiota and is considered a natural compound with clinical application value [7]. IPA can serve as an effective natural free radical scavenger *in vivo* and synergize with the well-known intracellular antioxidant GSH [8]. Therefore, IPA prevents oxidative damage to the liver [9]. Meanwhile, oral administration of IPA after intraperitoneal injection of lipopolysaccharide (LPS) can reduce pro-inflammatory cytokines, IL-1β, IL-6, and TNF-α [10]. The level of IFN-γ, IL-1β, and other pro-inflammatory cytokines play an important role in mediating APAP-induced liver injury[11]. However, its specific mechanism of action has not yet been elucidated. IPA plays an important role in regulating various immune cells throughout the body to maintain an anti-inflammatory microenvironment [7].

APAP-induced ALI remains a critical clinical challenge due to its high mortality and limited therapeutic options. Although gut microbiota-derived metabolites have been increasingly recognized for their role in liver homeostasis, the protective mechanisms of IPA, a tryptophan metabolite, against APAP-induced toxicity remain poorly understood. Here, we show that IPA levels are profoundly depleted in APAP-treated mice, correlating with aggravated liver injury and hepatocyte apoptosis. Importantly, IPA exerts its protective effects independently of gut microbiota, as evidenced by its efficacy in microbiota-depleted models. Moreover, IPA promotes hepatocyte proliferation and improves survival in models of lethal APAP overdose. These findings reveal a previously unrecognized role for IPA in ALI pathogenesis and highlight its potential as a therapeutic candidate targeting oxidative stress, apoptosis, and AhR-mediated inflammatory responses.

## Materials and Methods

### Animals and Mouse Models

C57BL/6 mice (6-8 weeks; 20-25 g) were obtained from Nanjing Medical University Laboratory Animal Center. Mice were acclimatized and fed for 3 days prior to the start of the experiment. To establish a mice model of ALI, mice received daily intragastric administration of IPA (100 mg/kg; HY-W015229, MCE, USA) or vehicle control for 5 consecutive days[12]. Mice were fasted overnight for 12 h on fresh bedding prior to APAP injection. APAP (Macklin, China) was dissolved in sterile saline at 55°C to prepare the APAP solution. The solution was then cooled to 37°C and administered intraperitoneally to mice at a dose of 300 mg/kg to induce acute liver injury or 500 mg/kg to induce liver failure. Mice received an intraperitoneal injection of CH223191 (20 mg/kg; HY-12684, USA) 4 h before IPA treatment. Mice receiving saline served as controls.

Experiment for oral IPA supplementation in antibiotic-pretreated mice. Conventional mice were orally gavaged with saline or IPA for 5 consecutive days, and during the last 3 days, mice received a cocktail of antibiotics (ABX) (ampicillin sodium salt (200 mg/kg, Macklin, Cat# A800429, China), metronidazole (200 mg/kg, Macklin, Cat# M813526, China), neomycin sulfate (200 mg/kg, Macklin, Cat# N814740, China), and vancomycin (200 mg/kg, Cat# V105495, Aladdin)). Mice receiving saline served as controls.

At the time of sacrifice, mice were ethically euthanized using isoflurane and inferior vena cava incision. To measure the levels of alanine aminotransferase (ALT), aspartate aminotransferase (AST), IPA and cytokines, serum was collected and analyzed. Liver tissues were excised for analysis of cell populations, extracting tissue protein, RNA, and cutting tissue sections. To measure the levels of TRP and TRH in the intestine, mouse feces were collected and analyzed. For all experiments, age-matched (6-8 weeks) and sex-matched mice housed and maintained under specific pathogen-free conditions were used. Mice were housed in ventilated cages at a standard temperature (22 ± 2°C), and 12 h of light-dark cycle, with free access to water and regular chow, except where otherwise specified for particular experimental models. All animals were treated according to the guidelines for the use of experimental animals and approved by the Institutional Animal Care and Research Advisory Committee of Nanjing Medical University (IACUC-2402002). All studies were reported in accordance with ARRIVE guidelines.

### Cell Culture

The HepG2 cell line was provided by Wuhan Pricella Company (China). The HepG2 cells were cultured in DMEM containing 10% fetal bovine serum (FBS) at 37°C in a 5% CO_2_ incubator, with passaging performed based on changes in cell confluence.

### Cell Viability Assay

Cell viability was assessed using the MTT assay. HepG2 cells were seeded at a density of 1×10^4 cells per well in a 96-well plate. IPA (25/50/100/200 ng/mL) was added or not for 1 h, followed by treatment with APAP (15 mM) or H_2_O_2_ (300 μM) for 24 h. MTT (15 mg/mL) was then added and incubated for 4 h. After discarding the supernatant, dimethyl sulfoxide (DMSO) was added to dissolve the formazan crystals, and the absorbance was measured.

### Cytotoxicity Assay by Lactate Dehydrogenase (LDH)

Cellular toxicity was evaluated by measuring lactate dehydrogenase (LDH) release into the culture medium due to cellular necrosis. The assay was performed using the Cytotoxicity Detection KitPLUS LDH (0474492600, Roche Diagnostics, Germany) following the manufacturer’s instructions. The percentage of cytotoxicity was calculated as: % cytotoxicity = ((Sample O.D. – Control O.D.) / (Positive control O.D. – Control O.D.)) × 100.

### ROS Quantification *in vitro* by Fluorescence Microplate Reader

Cells were seeded in a 96-well plate at a density of 2 × 10^4^ cells/well. The probe dihydroethidium (DHE, 2140299, Invitrogen, Thermo Fisher Scientific Inc.) was used at a concentration of 10 μM in HBSS medium supplemented with glucose (4.5 g/L) and glutamine (4 mM) to measure superoxide production. After 30 min of probe incubation, vehicle or APAP was added, and ROS production was measured using a fluorescence microplate reader (CLARIOstar Plus, BMG Labtech, Germany) for 2 h with excitation/emission filter pairs of 488/550–580 nm. DAPI staining (358/455–465 nm) was used to normalize for cell number. Each condition was run in triplicate, and antimycin A (10 μM) was used as a positive control.

### Intracellular GSH Detection

HepG2 cells (2 × 10^6^ cells per well) were cultured in a 6-well plate for 24 h. After treatment with IPA (100 ng/mL) for 18 h, the cells were further treated with APAP (15 mM) for 4 h. Intracellular GSH levels were measured using a commercially available kit (S0053, Beyotime, China) according to the manufacturer’s instructions.

### Detection of GSH and GSSG Levels

Liver tissue was homogenized and centrifuged at 500×g for 10 min at 4°C to collect the supernatant. The levels of GSH and GSSG were measured using a commercially available kit (S0053, Beyotime) according to the manufacturer’s instructions, and the results were calculated based on the kit's protocol.

### Apoptosis Detection

To assess apoptosis levels in liver tissue, mouse livers were first isolated and cut into 2–4 mm pieces. These pieces were then transferred to tubes containing collagenase IV (HY-E70005D, MCE, USA) and DMEM medium (Gibco). The samples were incubated at 37°C with continuous rotation for 40 min. After incubation, the resulting cell suspension was passed through a 70 μm filter and centrifuged at 50 × g for 10 min to collect the pellet of liver cells. Apoptosis levels were detected using a commercially available kit (C1062L, Beyotime) following the manufacturer’s instructions and analyzed by flow cytometry. For *in vitro* assessment of apoptosis in HepG2 cells, cells were digested with trypsin at specified time points, collected, and centrifuged at 500 × g for 5 min. The cells were then washed twice with PBS and apoptosis levels were detected using a commercially available kit (C1062L, Beyotime, China) following the manufacturer’s instructions, with flow cytometry used for final analysis.

### Quantitative Real-Time-PCR

Total RNA was purified from cells using TRIzol reagent (Invitrogen, USA) according to the manufacturer’s instructions. Reverse transcription into cDNA was performed using a Transcriptor First-Strand cDNA Synthesis kit (Roche, USA). Quantitative real-time PCR was performed using SYBR green (Roche) on a Step OnePlus Real-Time PCR System (Applied Biosystems, USA). Relative quantification was used to compute the fold changes in expression, and GAPDH served as the internal control. (Ahr Forward SequenceCACAGAGTTAGACCGCCTGG, Reverse primer GCCATTCAGCGCCATCAAAG).

### Western Blotting Analysis

First, liver tissue was obtained, and protein lysis buffer was added to lyse the tissue on ice for 30 min to extract total cellular protein. The protein concentration was quantified using the BCA method. 20 μg of protein was taken, denatured for 5 min, and subjected to SDS-PAGE electrophoresis at 80 V. The protein samples were then transferred to a membrane, blocked with 5% skim milk at room temperature for 2 h, and incubated with primary antibodies overnight at 4°C. The primary antibodies used were rabbit anti-CYP2E1 antibody (1:1000, ab28146), rabbit anti-Caspase3 antibody (1:1000, ab32042), rabbit anti-Cleaved Caspase3 antibody (1:2000, ab214430), rabbit anti-AHR (1:1000, ab308215), and mouse anti-β-actin antibody (1:1000, ab8226) (Abcam, UK). The secondary antibody used for immunoblotting was goat anti-rabbit/mouse IgG conjugated with horseradish peroxidase (1:5000, BL003A or BL001A, Biosharp). Immunoreactivity was evaluated using an enhanced chemiluminescence (ECL) detection kit (Solarbio Life Sciences, China), and the blots were analyzed using ImageJ (version 1.8.0).

### Flow Cytometric Analysis

Flow cytometry analysis was performed to assess the effect of IPA on inflammation in acute liver injury tissue. Briefly, non-parenchymal liver cells were isolated as previously described [13]. In short, the liver was perfused in situ with phosphate-buffered saline and homogenized, followed by density gradient centrifugation of the obtained single-cell suspension. The non-parenchymal cells were then stained with Pacific Orange succinimidyl ester (Thermo Fisher Scientific) to exclude dead cells and labeled with fluorescently conjugated antibodies against CD45 (110724), CD11b (101262), and Ly6G (127608) (all from BioLegend, USA). The analysis was conducted using BD FACS LSR II (BD Biosciences, USA) and FlowJo software (version 10.5.0; BD Biosciences).

### Enzyme-Linked Immunosorbent Assay (ELISA)

ALT (C009-2-1, Nanjing Jiancheng Bioengineering Institute, China) and AST (C010-2-1, Nanjing Jiancheng Bioengineering Institute) activities in the plasma were measured using commercial reagents according to the manufacturer’s instructions. Feces, serum or liver levels of IPA (ZC-53467-J, ZCIBIO, China), AhR (ml106143, mlbio, China), IFN-γ (MIF00-1, R&D Systems, USA), TNF-α (MTA00B-1, R&D Systems), IL-1β (MLB00C-1, R&D Systems), IL-6 (M6000B-1, R&D Systems) were measured using ELISA kits according to the manufacturer’s instructions.

### Hematoxylin and Eosin Staining and Immunofluorescence Staining

Mouse liver tissues were fixed in 4% formaldehyde, dehydrated using graded ethanol, and embedded in paraffin. After sectioning at 4-μm thickness, samples were subjected to hematoxylin and eosin staining. The degree of liver damage was evaluated according to Suzuki score. For histological analysis, liver sections from each animal were independently evaluated by investigators blinded to the experimental groups. At least three representative sections per liver were analyzed. Necrotic areas were quantified using ImageJ software and expressed as the percentage of necrotic area relative to the total tissue area. Suzuki scores were determined according to established criteria based on the degree of hepatocellular necrosis, congestion, and tissue injury. For immunostaining of liver, slices stained with primary antibodies (rabbit anti-Ki-67, ab15580, Abcam, 1:1000; rabbit anti-CD11b, ab133357, Abcam, 1:20000) diluted in 2% NDS / PBST overnight at 4? Sections were washed in PBST, then stained with secondary antibodies (donkey anti-rabbit Alexa594, ab150076, Abcam, 1:500; donkey anti-rabbit Alexa488, ab150061, Abcam, 1:500) diluted in 2% NDS / PBST for 2 h at RT. After washing with PBS, sections were imaged by an Olympus Fluoview 1000 confocal microscope. TUNEL staining was performed using a commercially available kit (G1516, Servicebio, China) according to the manufacturer’s instructions.

### Statistical Analysis

All data are shown as the mean ± SEM from at least three independent experiments. Significant differences were evaluated using one-way ANOVA or the Mann–Whitney U test with GraphPad Prism (version 9.3.1, GraphPad Software) and Statistical Package for Social Science software (version 22.0, SPSS). No sample was excluded from the analyses. Animals were not randomly assigned during collection, but the strain, sex, and age of the mice were the same, and the data analysis was single masked. Investigators were not blinded to the group allocation during the experiment and outcome assessment. The number of replicates and statistical method were indicated in each figure legend. Unless otherwise stated, all n values reported in this study represent biological replicates.

## Results

### IPA Is Significantly Decreased in a Mouse Model of APAP-Induced ALI

**Fig. 1:**
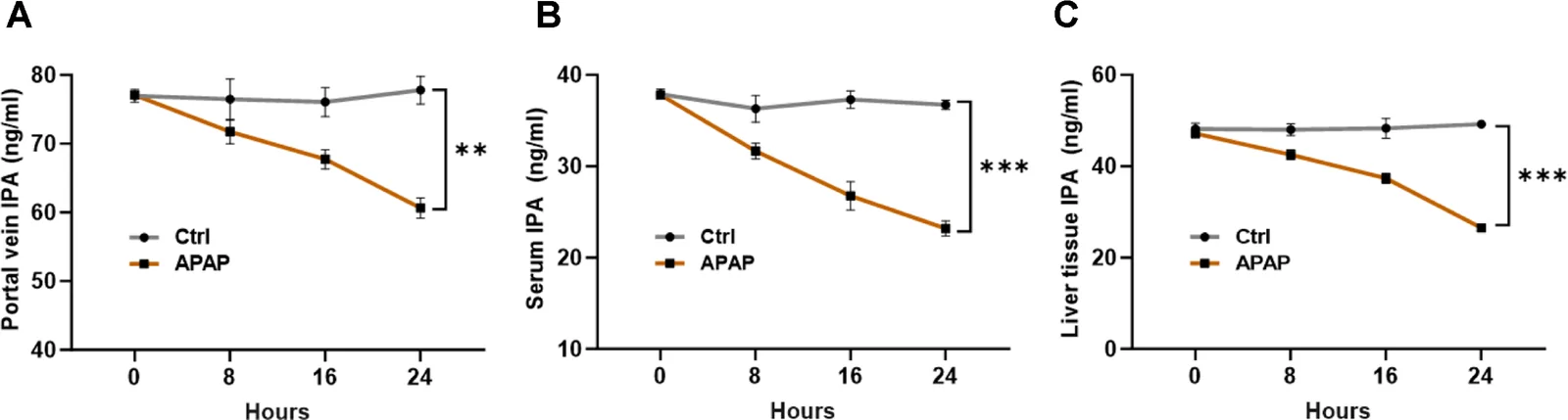
APAP-induced decrease of IPA in ALI mouse model. (**A-C**) IPA expression levels in the portal vein, serum, or liver tissues of C57BL/6 mice untreated or treated with APAP (n = 3 in each group). Data were presented as mean ± SEM. *p* values were calculated by one-way ANOVA with Tukey’s multiple comparisons in (**A**), (**B**) and (**C**); *p < 0.05, **p < 0.01, ***p < 0.001.

As IPA is an important product of tryptophan metabolism by intestinal flora and an effective natural free radical scavenger. We evaluated changes in indole-3-propionic acid (IPA) levels in the portal vein, liver, and serum of APAP-treated mice at different time points (0, 8, 16, and 24 h). Using C57BL/6 mice as a model of APAP-induced liver injury, we found that IPA levels in the portal vein, serum, and liver tissues were significantly decreased in APAP-treated mice (Fig. 1A–1C). These findings suggest that APAP-induced acute liver injury leads to a global decrease in IPA metabolic levels in mice.

### IPA Enhances Cell Viability and Inhibits APAP-Induced Apoptosis

**Fig. 2:**
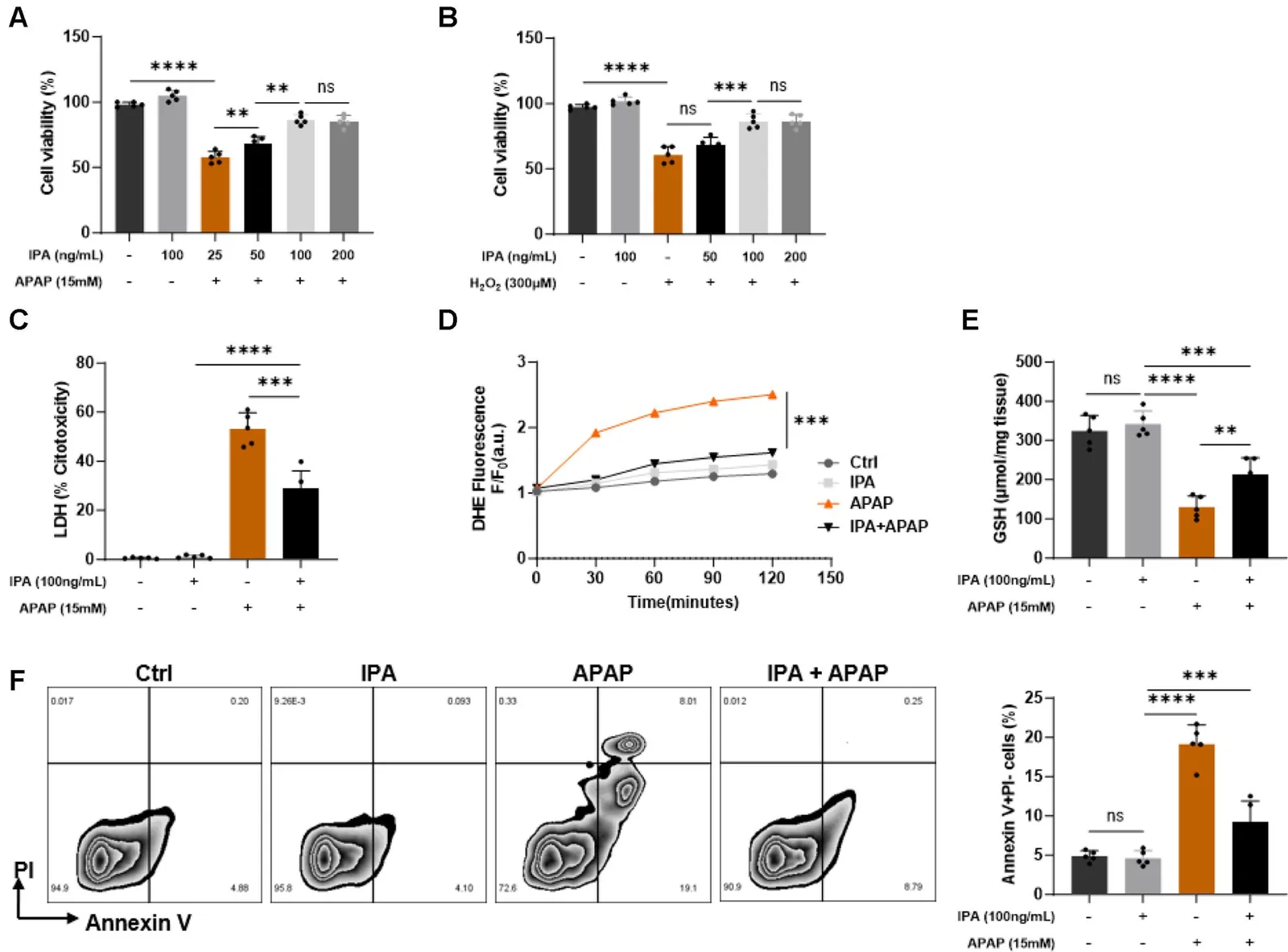
Effects of IPA treatment on APAP-induced cytotoxicity and apoptosis in HepG2 cells. (**A-B**) HepG2 cells were treated with IPA for 24 h in the absence or presence of APAP or H_2_O_2_, and cell viability was assessed using the MTT assay (n=5 in each group). (**C**) Cytotoxicity determined by lactate dehydrogenase (**LDH**) release. Data are presented as percentage relative to the positive control (100%). (**D**) ROS production detected with DHE probe represented as DHE fluorescence F/F_0_ (a.u.) through exposure to APAP during 2 h. (**E**) The levels of GSH in the cells from the groups in (**A**) were measured (n = 5 in each group). (**F**) Annexin/PI levels in the cells from the groups in (**A**) were analyzed by flow cytometry (n = 5 in each group). Data were presented as mean ± SEM. *p* values were calculated by one-way ANOVA with Tukey's multiple comparisons in (**A**), (**B**), (**C**), (**D**), (**E**) and (**F**); *p < 0.05, **p < 0.01, ***p < 0.001, ****p < 0.0001, ns, not significant.

First, we sought to determine whether IPA exerts a protective effect against APAP or H_2_O_2_-induced cytotoxicity in vitro. The MTT assay was used to analyze changes in HepG2 cytotoxicity induced by APAP or H_2_O_2_. The results showed that IPA treatment significantly suppressed APAP- or H_2_O_2_-induced cytotoxicity in HepG2 cells (Fig. 2A and 2B). However, when the concentration of IPA reached 100 ng/mL, no further enhancement in the protective effect was observed; therefore, subsequent experiments were conducted using 100 ng/mL IPA. Furthermore, compared with the control group cells, we detected a significant decrease in lactate dehydrogenase (LDH) release in APAP-exposed HepG2 cells treated with IPA (Fig. 2C). Subsequently, we used the dihydroethidium (DHE) probe to detect the production of reactive oxygen species (ROS) in these cells within 2 hours after APAP stimulation, and found that APAP-induced ROS were barely observed in IPA-treated HepG2 cells (Fig. 2D). IPA (100 ng/mL) treatment significantly inhibited APAP-induced GSH depletion (Fig. 2E). Additionally, flow cytometry analysis demonstrated that IPA treatment significantly suppressed APAP-induced apoptosis (Fig. 2F).

### IPA Significantly Alleviates Liver Injury in a Mouse Model of APAP-Induced ALI

**Fig. 3:**
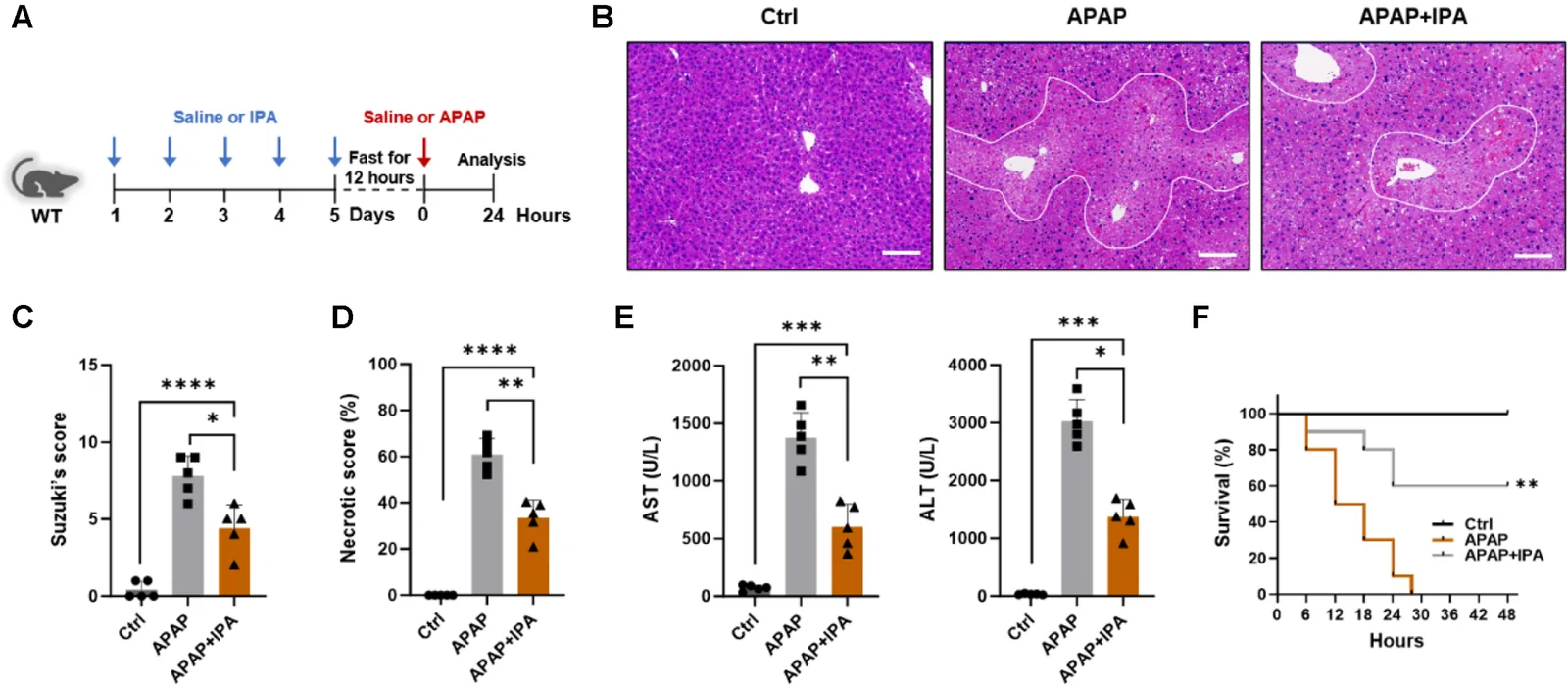
IPA significantly reduces liver damage in ALI mouse model. (**A**) Schematic illustration of the experimental procedure. C57BL/6 mice were gavaged once daily with or without IPA for 5 days, followed by 12-h fasting. Mice then received APAP or vehicle via intraperitoneal injection, and tissue samples were collected 24 h later. (**B**) H&E staining of liver tissues from APAP-induced C57BL/6 mice treated with IPA or saline (n = 5 in each group). Scale bar = 100 μm. (**C–D**) Quantitative analysis of necrotic areas and Suzuki’s scores in liver tissues of APAP-induced C57BL/6 mice treated with IPA or saline (n = 5 in each group). (**E**) Serum ALT and AST levels in APAP-induced C57BL/6 mice treated with IPA or saline (n = 5 in each group). (**F**) Survival curves of APAP-induced C57BL/6 mice treated with IPA or saline (n = 10 in each group). Data are presented as mean ± SEM. *P* values were calculated using one-way ANOVA followed by Tukey’s multiple comparisons test in (**C**), (**D**), and (**E**), and the log-rank (Mantel–Cox) test in (**F**). *p < 0.05, **p < 0.01, ***p < 0.001, ****p < 0.0001.

To investigate the specific role of IPA in APAP-induced liver injury, we used C57BL/6 mice with or without IPA gavage once daily for 5 days, followed by the establishment of an APAP-induced ALI model (Fig. 3A). APAP-induced liver tissue damage was confirmed by observing H&E-stained sections (Fig. 3B) and assessing the Suzuki score, necrotic areas, and ALT and AST levels. IPA treatment also significantly reduced the Suzuki score and necrotic area in mice compared with those in control mice (Fig. 3C and 3D), and serum ALT and AST levels were significantly reduced in IPA-treated mice (Fig. 3E). To determine whether IPA treatment protected mice from death due to APAP overdose, WT mice were injected with a higher dose of APAP (500 mg/kg), and after 48 h, 100% of WT mice died from a lethal dose of APAP, compared with 60% survival in IPA-treated mice (Fig. 3F). These results indicated that IPA treatment significantly improved the survival rate of mice administered with an excessive dose of APAP. Collectively, IPA exerted significant protective effects against APAP-induced ALI.

### IPA Markedly Alleviates Liver Injury in Microbiota-Depleted Mice with APAP-Induced ALI

To investigate the specific role of IPA derived from gut microbiota in APAP-induced liver injury, mice were orally treated with IPA while microbiota-depleted mice were generated using antibiotics (ABX), and subsequently an ALI model was established (Fig. 3A). APAP-induced liver tissue damage was confirmed by H&E staining (Fig. 3B) and by assessing Suzuki scores, necrotic areas, and serum ALT and AST levels. IPA treatment significantly reduced the Suzuki scores and necrotic areas in both wild-type (WT) and microbiota-depleted mice compared with control mice (Fig. 3C and 3D), and serum ALT and AST levels were significantly decreased in IPA-treated mice (Fig. 3E). To determine whether IPA treatment protected mice from death due to APAP overdose, WT mice were injected with a higher dose of APAP (500 mg/kg), and after 48 h, 100% of WT mice succumbed to the lethal dose, whereas 40% of IPA-treated mice survived (Fig. 3F). These results indicated that IPA treatment significantly improved the survival rate of mice administered a lethal dose of APAP, suggesting that IPA exerts a significant protective effect against ALI in mice.[Fig F4]

### IPA Significantly Reduced APAP-Induced Cell Apoptosis

**Fig. 5:**
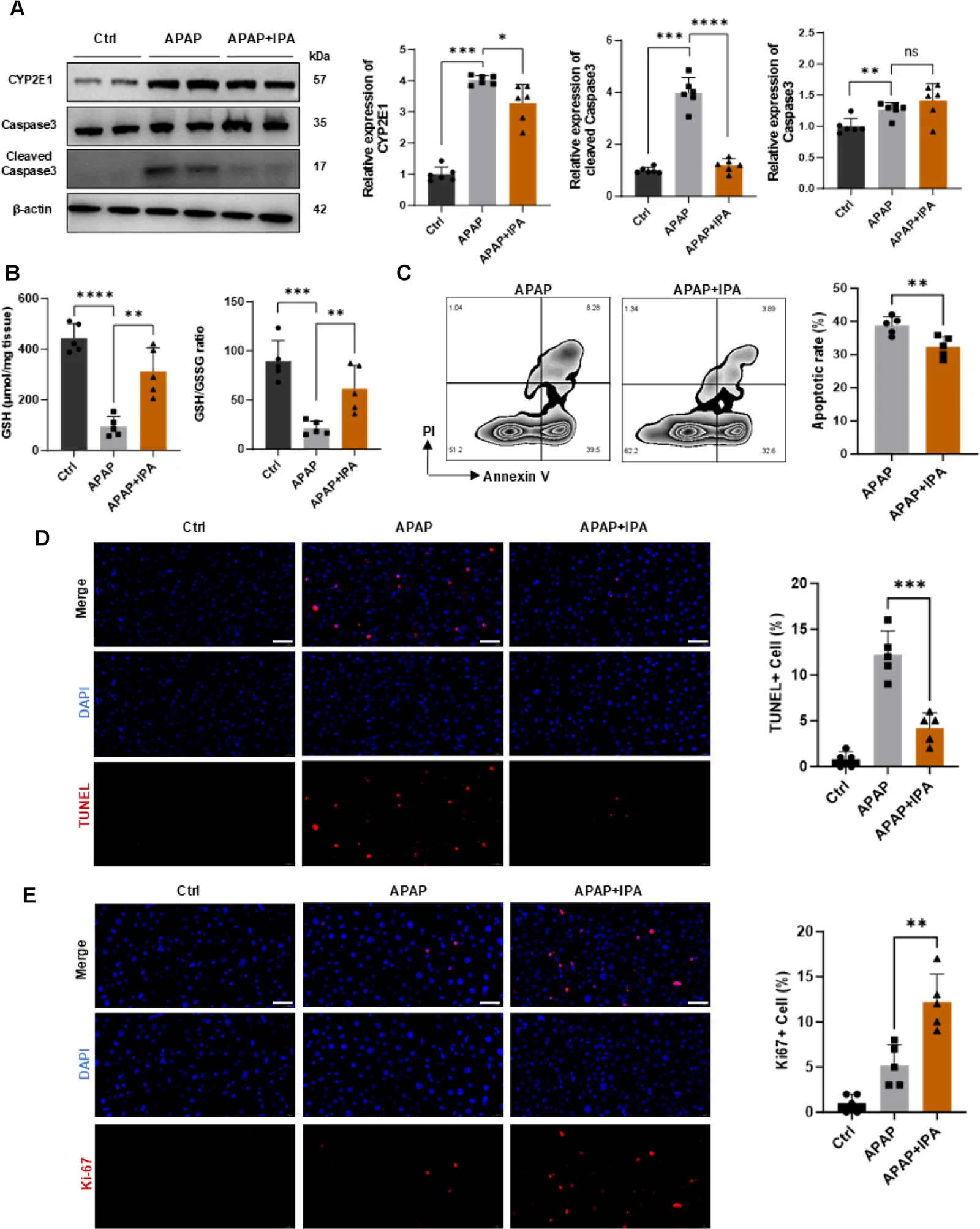
IPA significantly reduced APAP-induced cell apoptosis. (**A**) Western blot and quantitative analysis of CYP2E1, Caspase-3 and Cleaved-caspase-3 in liver tissues of APAP or saline treated mice treated with IPA or saline (n = 6 in each group). (**B**) Expression levels of GSH and GSH/GSSG in liver tissues of APAP or saline treated mice treated with IPA or saline (n = 5 in each group). (**C**) Flow cytometry analysis and statistical analysis of Annexin V/PI expression ratio in hepatocytes of APAP or saline treated mice treated with IPA or saline (n=5 in each group). (**D**) Representative images and quantitative analysis of TUNEL immunofluorescence staining of liver tissue of APAP or saline treated mice treated with IPA or saline (n = 5 in each group), scale bar represents 20 μm (**E**) Representative images and quantitative analysis of Ki-67 immunofluorescence staining of liver tissue of APAP or saline treated mice treated with IPA or saline (n = 5 in each group), scale bar represents 20 μm. ns, not significant; Data were presented as mean ± SEM. *p* values were calculated by one-way ANOVA with Tukey’s multiple comparisons in (**A**), (**B**), (**C**), (**D**) and (**E**); *p < 0.05, **p < 0.01, ***p < 0.001, ****p < 0.0001, ns, not significant.

APAP overdose induces hepatotoxicity, which ultimately leads to hepatocyte apoptosis [14]. Next, we explored whether IPA enhances APAP clearance and mitigates apoptotic responses in hepatocytes. C57BL/6 mice were treated with APAP to establish an ALI model, and IPA or saline was orally administered for 6 h. Western blot analysis showed that IPA treatment significantly inhibited CYP2E1 protein expression compared with that in the control (Fig. 5A). Additionally, IPA-treated mice had significantly reduced expression of the cell death marker, cleaved-caspase-3 in the liver tissue (Fig. 5A). GSH protects the liver from oxidative stress [15]. IPA treatment significantly increased GSH expression and the ratio of GSH to reduced glutathione (Fig. 5B). Flow cytometry was used to detect Annexin V/PI and evaluate hepatocyte apoptosis (Fig. 5C). IPA treatment reduced the rate of apoptosis in hepatocytes. Immunofluorescence analysis of APAP-stimulated liver tissue at 6h showed that TUNEL staining was significantly decreased in the IPA group compared with that in the control group (Fig. 5D), while Ki-67 staining was significantly increased in the IPA group (Fig. 5E). These results indicate that IPA intervention significantly reduced APAP-induced hepatocyte apoptosis and enhanced APAP metabolism and hepatocyte proliferation.

### IPA Inhibits APAP-Induced ALI and Is Related to Inflammatory Cells

**Fig. 6:**
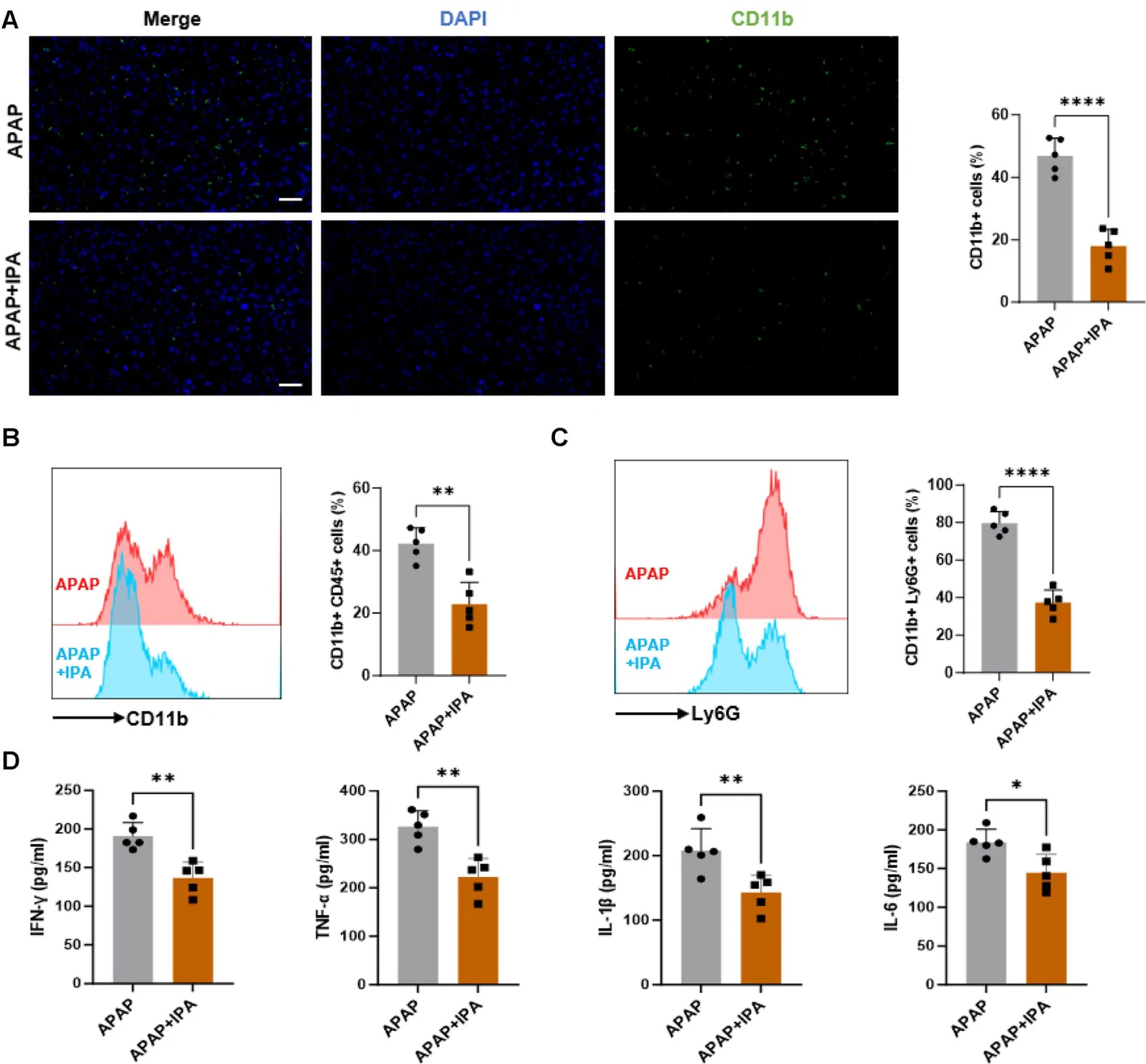
IPA Inhibits Inflammatory Cell Infiltration in APAP-Induced ALI. (**A**) Representative images and quantitative analysis of CD11b immunofluorescence staining of liver tissue of APAP or saline treated mice treated with IPA or saline (n = 5 in each group), scale bar represents 50 μm. (**B**) Flow cytometry analysis and statistical analysis of proportion of CD45+CD11b+ cells in ALI of APAP treated mice treated with IPA or saline (n = 5 in each group). (**C**) Flow cytometry analysis and statistical analysis of proportion of CD45+ Ly6G+ cells in ALI of APAP treated mice treated with IPA or saline (n = 5 in each group). (**D**) Expression levels of cytokines IFN-γ, TNF-α, IL-6, IL-1β of liver tissue of APAP or saline treated mice treated with IPA or saline (n = 5 in each group). Data were presented as mean ± SEM. *P* values were calculated by two-tailed Student’s t test in (**A**), (**B**), (**C**) and (**D**); *p < 0.05, **p < 0.01, ***p < 0.001, ****p < 0.0001.

Studies have shown that the severity of APAP-induced ALI is critically dependent on the infiltration of inflammatory monocytes and neutrophils. Whether IPA pretreatment inhibits APAP-induced liver injury in relation to inflammatory monocytes was evaluated. Immunofluorescence results showed that, compared to control mice treated with saline and APAP, the liver of IPA-treated ALI mice had significantly lower levels of inflammatory monocytes and neutrophil marker CD11b (Fig. 6A). Moreover, flow cytometry confirmed that compared to the APAP-treated control mice, the accumulation of CD11b+ cells in the liver was reduced (Fig. 6B), with Ly6G+ neutrophils also significantly reduced (Fig. 6C). Better recovery of liver tissue in the ALI group was attributed to the effective resolution of inflammation; therefore, the inflammation of liver tissue in each group was further evaluated. ELISA results showed that IPA treatment significantly reduced serum pro-inflammatory cytokines, IFN-γ, TNF-α, IL-6, and IL-1β levels in mice compared with those in the control group (Fig. 6D), contributing to the regression of inflammation. In conclusion, IPA can significantly improve the inflammatory state of APAP-induced ALI.

### IPA Inhibits APAP-Induced ALI through AhR

**Fig. 7:**
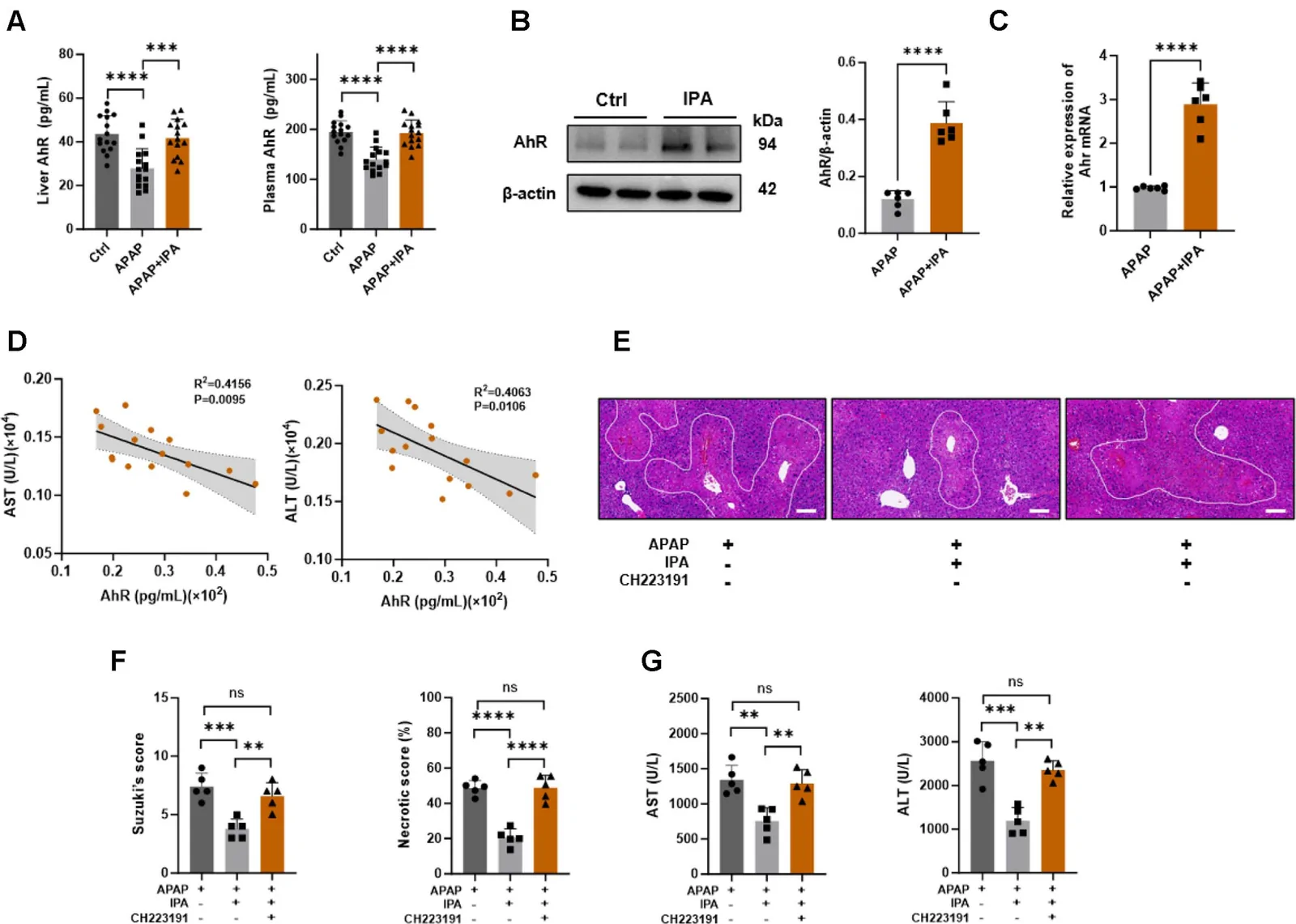
IPA inhibits APAP-induced ALI injury through AhR. (**A**) ELISA was used to detect the expression levels of AhR in the liver tissue of mice treated with saline, APAP, or IPA (n = 15 in each group). (**B**) ELISA was used to detect the expression levels of AhR in the serum of mice treated with saline, APAP, or IPA (n = 15 in each group). (**C**) Western blot analysis of the expression levels of AhR in the liver tissue of mice treated with APAP, or IPA (n = 6 in each group). (**D**) Correlation analysis between liver AhR levels and serum AST and ALT levels. (**E**) H&E staining of liver tissues of APAP-induced C57BL/6 mice treated with APAP, IPA or CH223191 (n = 5 in each group), scale bar represents 100 μm. (**F**) Suzuki's score and quantitative analysis of necrotic regions in liver tissues of APAP-induced C57BL/6 mice treated with APAP, IPA or CH223191, (n = 5 in each group). (**G**) Serum ALT/AST expression levels of APAP-induced C57BL/6 mice treated with APAP, IPA or CH223191 (n = 5 in each group). Data were presented as mean ± SEM. *p* values were calculated by one-way ANOVA with Tukey’s multiple comparisons in (**A**), (**F**) and (**G**); two-tailed Student’s t test in (**B**) and (**C**), and Spearman’s rank-order correlation in (**D**); *p < 0.05, **p < 0.01, ***p < 0.001, ****p < 0.0001, ns, not significant.

IPA exerts its regulatory function through AhR [16]. Therefore, we further investigated the AhR levels in the liver of APAP-induced ALI mice treated with IPA. ELISA results showed that compared to the saline treatment group, AhR levels in the liver and serum of APAP-induced ALI mice were significantly decreased, but IPA treatment restored AhR expression (Fig. 7A). Western blot analysis revealed that, compared to the APAP treatment group, IPA treatment significantly enhanced the expression of AhR protein and its mRNA (*Ahr*) in the liver (Fig. 7B and 7C). To further investigate whether AhR can serve as a potential biomarker for APAP-induced acute liver toxicity, we performed correlation analysis between liver AhR levels and serum AST and ALT levels. The results showed that in APAP-induced ALI mice, the levels of AhR were negatively correlated with both AST and ALT levels (Fig. 7D), indicating that higher AhR levels corresponded to lower AST and ALT levels. This suggests that AhR plays a protective and predictive role in APAP-induced ALI. We continued to verify the important role of AhR through *in vivo* experiments. CH223191 is an inhibitor of AhR. Compared to the APAP control group, IPA treatment significantly reduced the Suzuki score and necrotic area in mice (Fig. 7E and 7F), and serum ALT and AST levels were significantly reduced in IPA-treated mice (Fig. 7G). However, the aforementioned phenomena were inhibited by pre-treatment with CH223191. In conclusion, IPA alleviates the severity of APAP-induced ALI through AhR.

## Discussion

Exploring the relationship between the gut microenvironment and liver disease has always been a popular international research topic. Gut microbiota can regulate liver immunity and metabolism by secreting various metabolites that affect disease progression. Multiple pieces of evidence suggest that excessive APAP leads to liver injury accompanied by dysbiosis of the gut microbiota and barrier damage [17], which further leads to an increase or decrease in the corresponding metabolites. The intestinal tryptophan metabolite IPA plays a role in maintaining intestinal epithelial homeostasis in intestinal injury models [18]. It also inhibited intestinal dysbiosis and endotoxin leakage, thereby reducing steatohepatitis in rats [19]. In this study, we demonstrated that IPA could alleviate liver damage caused by excessive APAP.

In APAP-induced ALI models, the intestinal microflora has an important but unknown function. Dysregulated gut microflora in mice with APAP-induced liver injury can act as a risk factor for transfer to healthy WT mice [17], suggesting that dysregulated gut microflora can contribute to the progression of liver injury. Additionally, the important components of the intestinal flora that affect liver diseases are the metabolites of the flora. Intestinal flora inhibits APAP-induced hepatotoxicity by secreting metabolites, such as phenyl propionic acid and daidzein [20, 21]. However, the specific effects of the intestinal flora metabolites on APAP-induced liver injury remain unclear. IPA has important organ-protective effects, including the reduction of tissue inflammation [22]. In this study, we observed a significant decrease in the ability of APAP-induced ALI mice to produce IPA. IPA, a gut microbiota-derived metabolite of tryptophan, exerts a key role in this process by functioning as a potent antioxidant and anti-inflammatory molecule [23].

IPA mitigated APAP/H_2_O_2_-induced cytotoxicity by scavenging ROS and restoring GSH levels, consistent with its role as a free radical scavenger [24]. These findings are in line with previous studies showing that IPA inhibits lipid peroxidation and preserves mitochondrial integrity in hepatic models. In addition, HepG2 cells were used for the *in vitro* experiments. Although widely used in studies of liver injury, HepG2 cells do not fully reflect the metabolic characteristics of primary hepatocytes, particularly their CYP-mediated drug metabolism. Therefore, future studies using primary hepatocytes or other physiologically relevant models are needed to further validate the protective effects of IPA against APAP-induced hepatotoxicity. To investigate the specific hepatoprotective effects of IPA, APAP-induced ALI models were established in both wild-type and microbiota-depleted mice. Moreover, IPA suppressed apoptosis by downregulating cleaved caspase-3 and reducing TUNEL-positive cells, mirroring observations in NASH where IPA attenuates hepatocyte death via NF-κB inhibition [19]. IPA treatment improved liver inflammation, reduced hepatocyte death, decreased CYP2E1 expression, and increased GSH levels, suggesting enhanced APAP metabolism and cytoprotection. Collectively, these results indicate that IPA effectively protects against APAP-induced liver injury.

Mechanistically, IPA attenuated inflammatory responses by reducing hepatic infiltration of CD11b+ monocytes and Ly6G+ neutrophils, and decreasing serum pro-inflammatory cytokines including TNF-α, IL-6, IL-1β, and IFN-γ, supporting previous reports that microbial metabolites can modulate liver inflammation[25]. Furthermore, our findings suggest that activation of the aryl hydrocarbon receptor (AhR) may contribute, at least in part, to the hepatoprotective effects of IPA, as pharmacological inhibition of AhR with CH223191 attenuated the beneficial effects of IPA. Correlation analysis revealed a negative relationship between hepatic AhR levels and serum ALT/AST, indicating that AhR may serve as both a mediator and biomarker of APAP-induced liver injury. Collectively, our findings identify IPA as a critical gut microbiota–derived metabolite that alleviates APAP-induced ALI through antioxidative, anti-apoptotic, and anti-inflammatory effects, with AhR signaling potentially contributing, at least in part, to its hepatoprotective actions. These findings suggest that the IPA–AhR axis may represent a potential target for drug-induced liver injury. However, since IPA was administered prior to APAP exposure in this study, the observed effects primarily reflect a preventive strategy, and further studies are needed to determine whether IPA also exhibits therapeutic efficacy after APAP overdose.

## Conclusion

This study demonstrates that APAP exposure leads to a significant reduction in the gut microbiota-derived metabolite IPA, which may contributor to the progression of acute liver injury. Restoration of IPA levels confers robust hepatoprotective effects both *in vitro* and *in vivo* by attenuating oxidative stress, maintaining glutathione homeostasis, suppressing hepatocyte apoptosis, and improving survival following APAP overdose. Mechanistically, IPA mitigates inflammatory cell infiltration and cytokine production, and these protective effects may be partially mediated through activation of the AhR signaling pathway. Collectively, these findings identify IPA as a critical gut microbiota-derived metabolite that links intestinal dysbiosis to APAP-induced hepatotoxicity and suggest that the IPA–AhR axis may represent a potential therapeutic target for drug-induced acute liver injury.

## Figures and Tables

**Fig. 4 F4:**
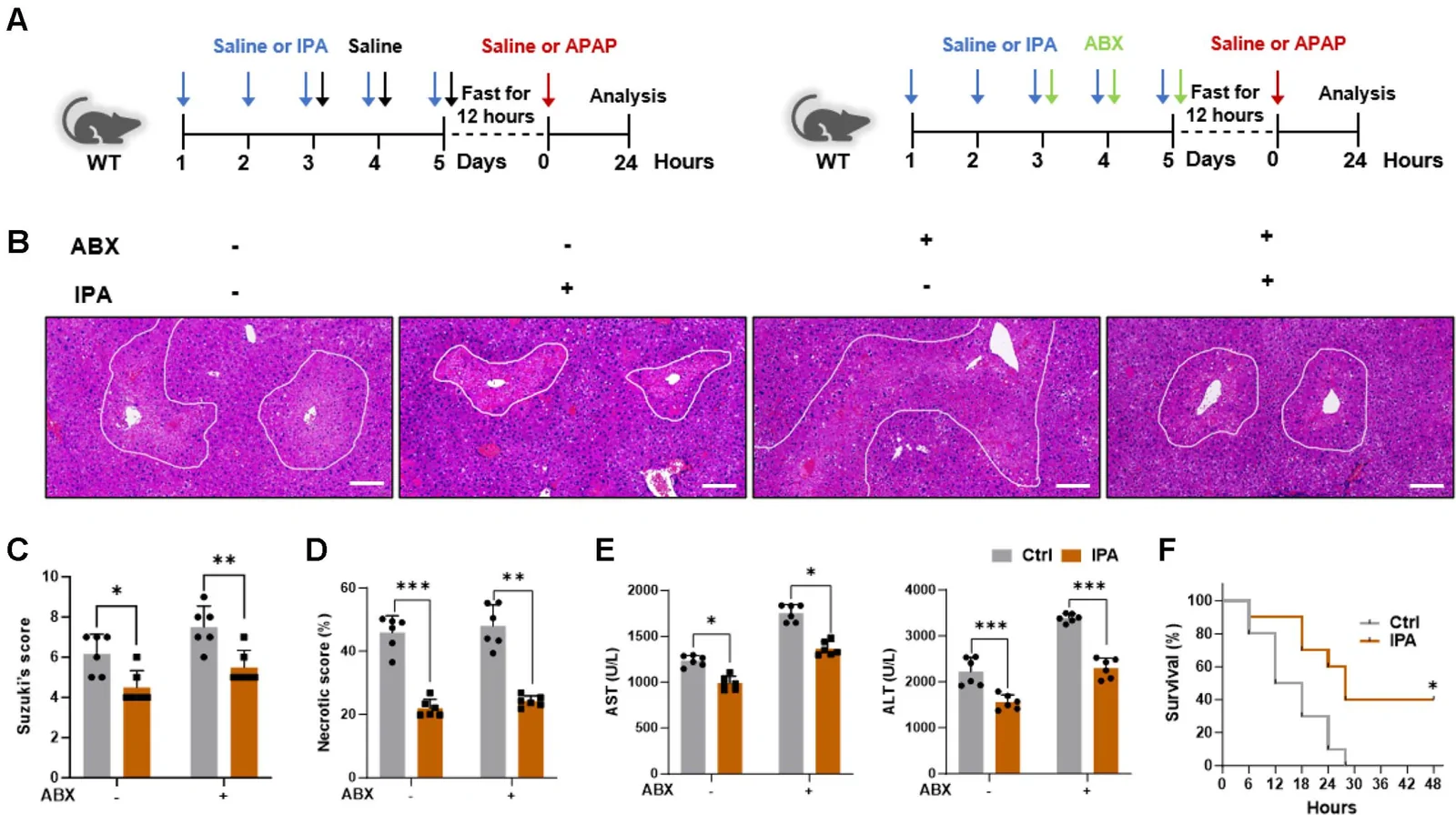
IPA significantly reduces liver damage in ALI mouse model. (**A**) Schematic illustration of the experimental procedure. C57BL/6 mice were orally gavaged with saline or IPA once daily for 5 days. Starting on day 3, mice received saline or antibiotics (**ABX**), followed by 12-h fasting. Mice were then intraperitoneally injected with APAP or vehicle, and tissue samples were collected 24 h later. (**B**) H&E staining of liver tissues from APAP-induced C57BL/6 mice treated with IPA or saline (n = 5 in each group). Scale bar = 100 μm. (**C-D**) Quantitative analysis of necrotic areas and Suzuki’s scores in liver tissues of APAP-induced C57BL/6 mice treated with IPA or saline (n = 5 in each group). (**E**) Serum ALT and AST levels in APAP-induced C57BL/6 mice treated with IPA or saline (n = 5 in each group). (**F**) Survival curves of APAP-induced C57BL/6 mice treated with IPA or saline (n = 10 in each group). Data are presented as mean ± SEM. *P* values were calculated using one-way ANOVA followed by Tukey’s multiple comparisons test in (**C**), (**D**), and (**E**), and the log-rank (Mantel–Cox) test in (**F**). *p < 0.05, **p < 0.01, ***p < 0.001, ****p < 0.0001.
